# Supplementary material for: Molecular mechanisms of Bdp1 in TFIIIB assembly and RNA polymerase III transcription initiation
Source: Nat Commun. 2017 Jul 25;8:130. doi: 10.1038/s41467-017-00126-1 (PMC5526994; doi:10.1038/s41467-017-00126-1)
Supplement: Supplementary file 1 — Supplementary Information [file 41467_2017_126_MOESM1_ESM.pdf]

File name: Supplementary Information
Description: Supplementary figures and supplementary table.

File name: Peer review file
Description:

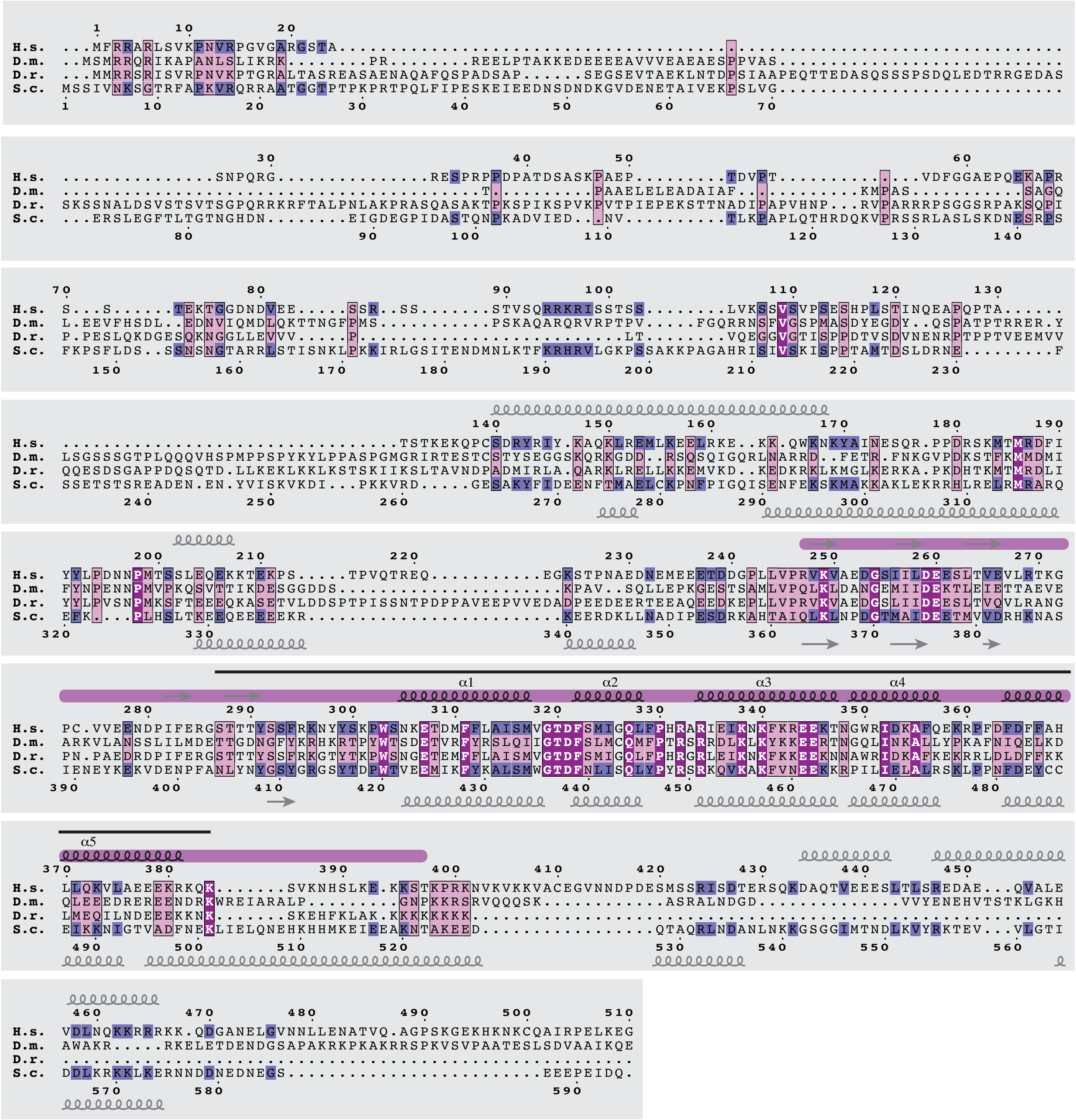

Supplementary Figure 1 Bdp1 sequence and architecture conservation.

Sequence alignment of the first 510 residues of *Homo sapiens* (H.s.) Bdp1 with *Drosophila melanogaster* (D.m.), *Danio rerio* (D.r.) and full-length *Saccharomyces cerevisiae* (S.c) Bdp1 proteins. Secondary structure predictions for H.s and S.c Bdp1 are depicted in gray above and below the sequence alignment, respectively. Structural elements observed in the TFIIIB-DNA structures are depicted in black. The purple line defines the boundary of the Bdp1 construct which crystallized as part of the TFIIIB-DNA complex and the black line indicates the region for which an atomic model could be built.

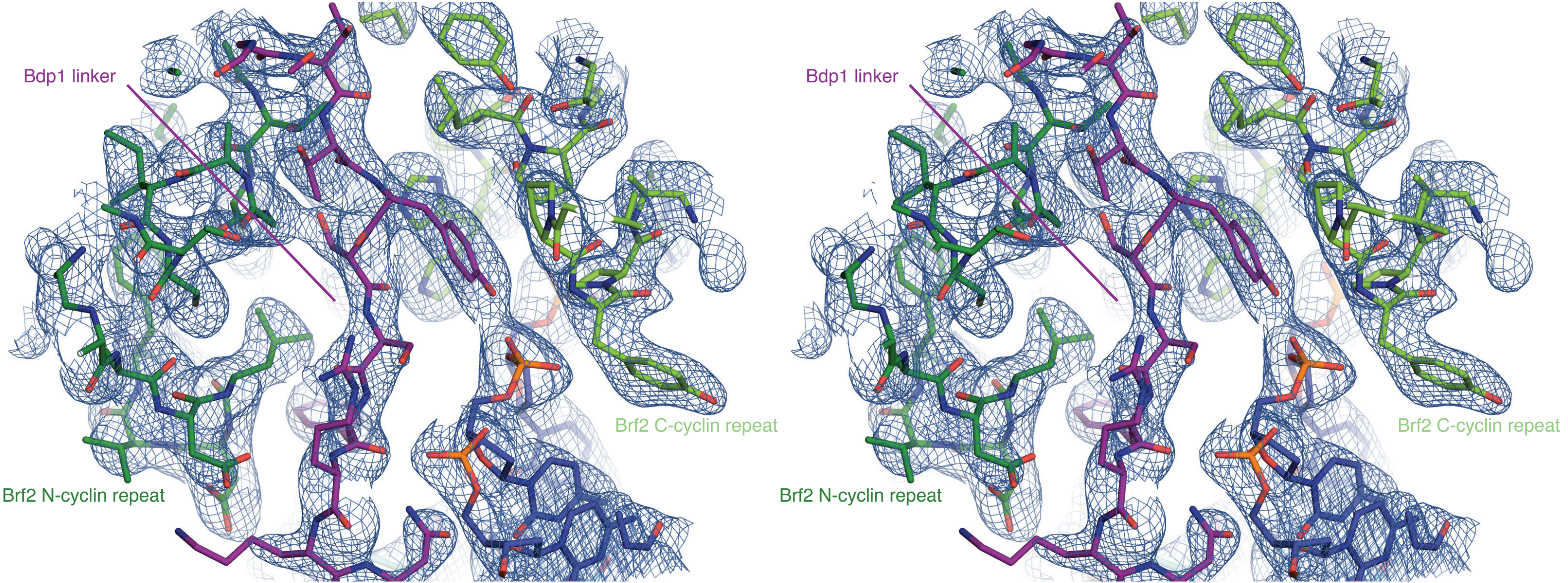

Supplementary Figure 2 Stereo close-up view of the Bdp1 linker and its interaction with Brf2 (green) and the DNA template (blue). The $2F_o$-F_c electron density maps is contoured at 1σ.

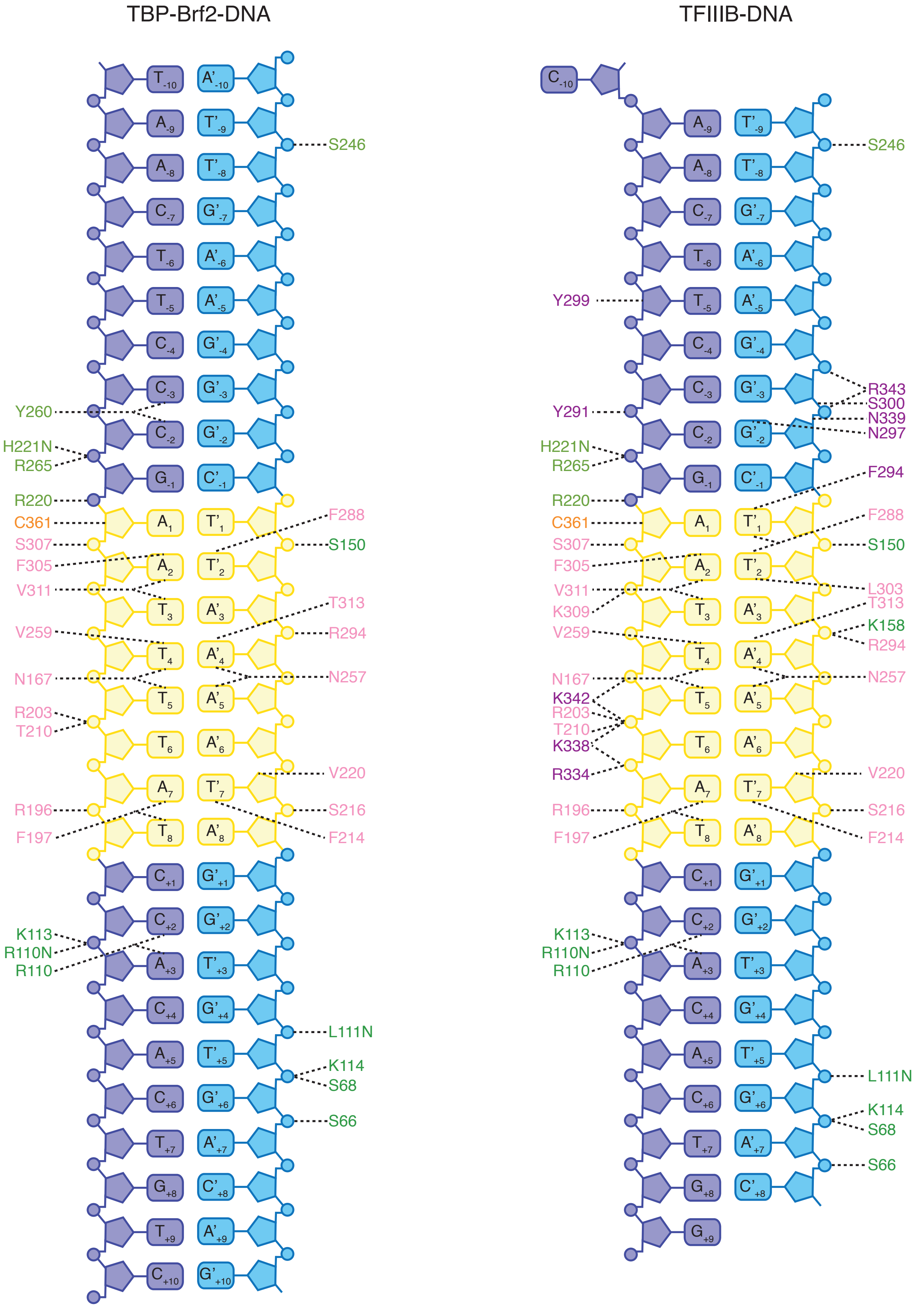

Supplementary Figure 3 Protein-DNA contacts in the Brf2-TBP-DNA structure (left) and in the TFIIIB-DNA structure (right). The template and non-template strands of the DNA are depicted in blue and cyan, respectively. The TATA box is indicated in yellow. Residues are color coded with Brf2 cyclin folds in green, Brf2 C-terminal domain in orange, TBP in pink and Bdp1 in magenta.

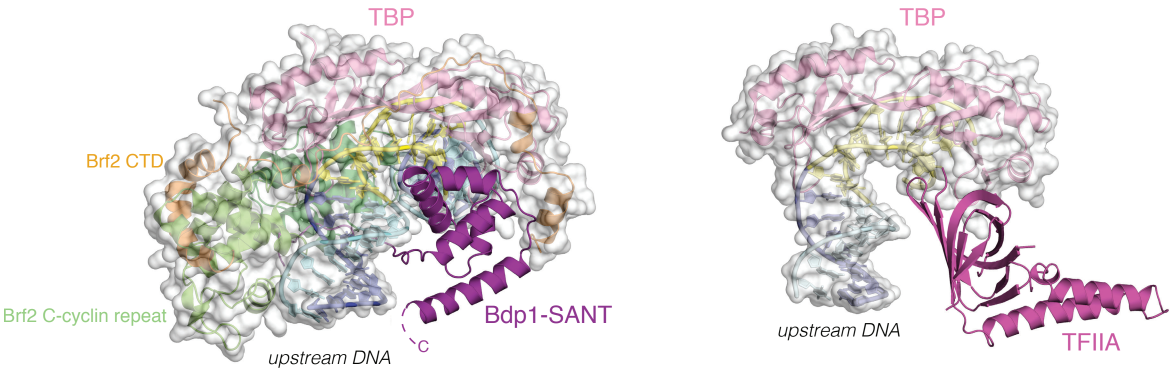

Supplementary Figure 4 Structural comparison of Bdp1 and TFIIA (PDB id: 1NH2)[22]. Color-coding as in Supplementary Fig. 3.

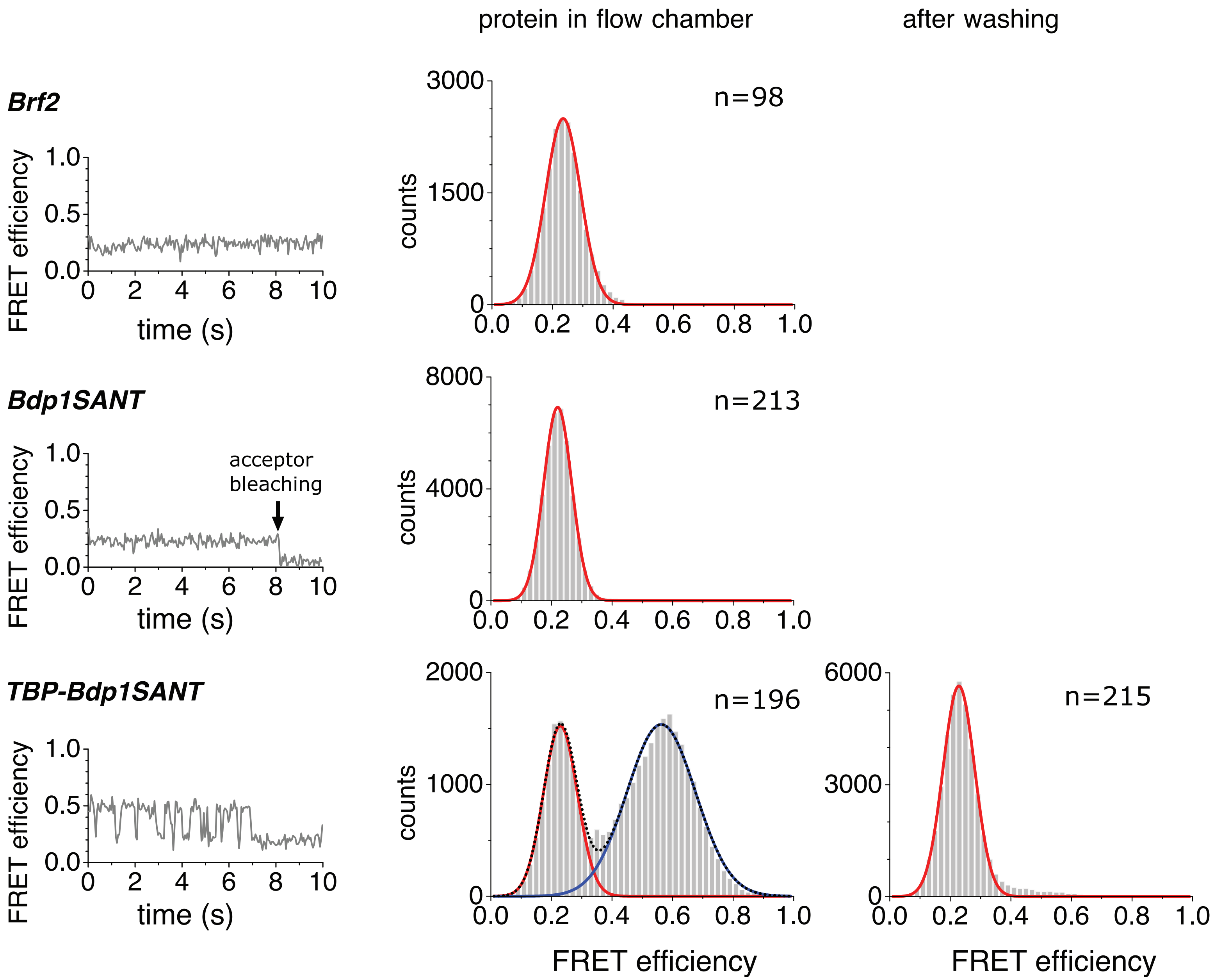

Supplementary Figure 5 Control measurements using Brf2, Bdp1SANT or Brf2 and Bdp1SANT have no effect on the FRET efficiency distribution and hence do not influence the U6 promoter DNA conformation. Bdp1SANT alone cannot stabilize TBP on the promoter. Single-molecule FRET measurements were carried out with a synthetic U6 snRNA promoter as shown in Fig. 1a. Measurements on immobilized molecules were conducted once with the proteins present in the flow chamber and after a washing step that removes freely diffusing proteins. The total number of molecules used for each plot is given as n. Each histogram was fitted with a single or double Gaussian distribution to determine mean FRET-efficiencies (see Supplementary Table 1).

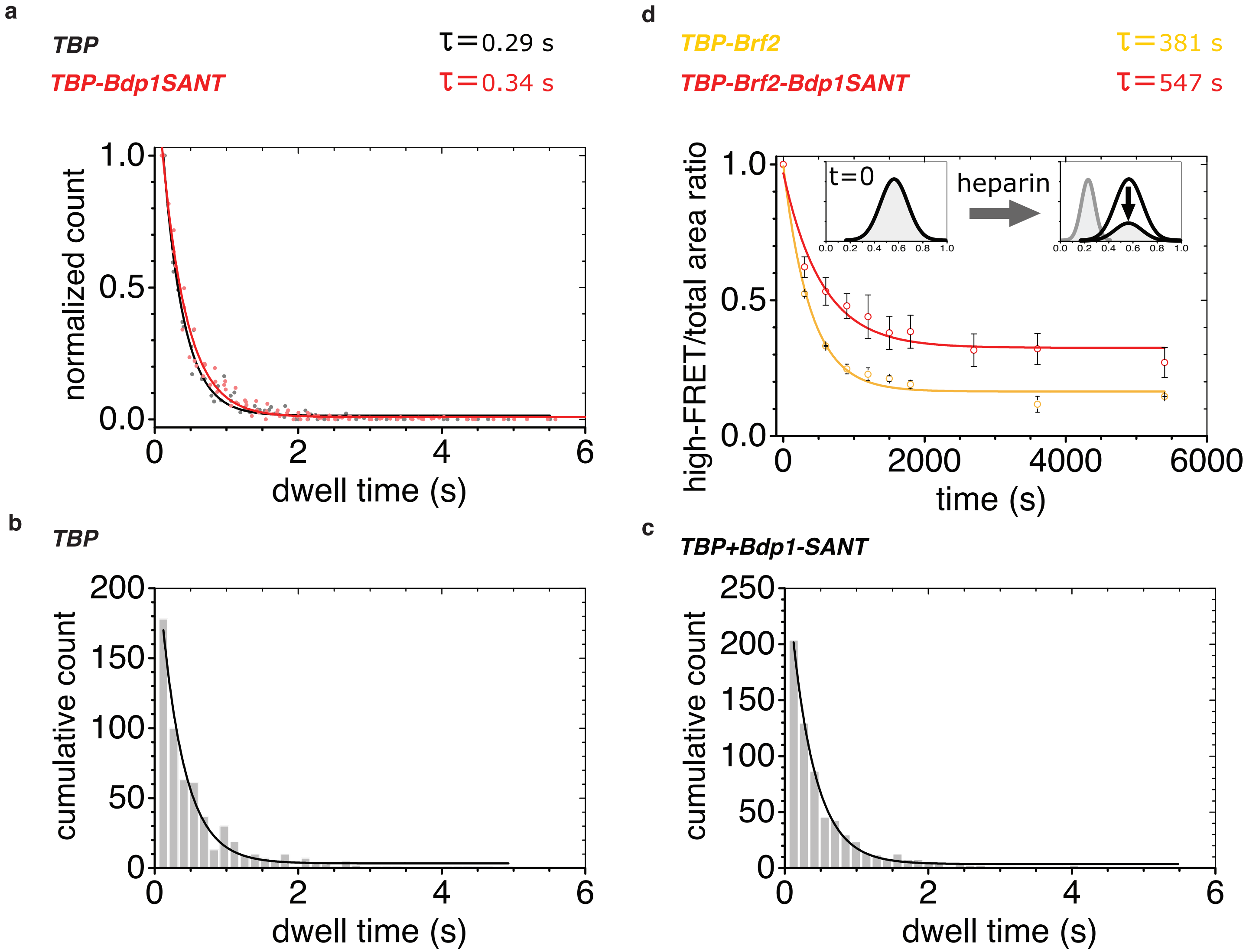

Supplementary Figure 6 Determination of complex lifetimes for DNA-TBP (fast dynamics) and DNA-TBP-Brf2 complexes. (a) Normalized dwell time histograms derived from dynamic single-molecule transients of the promoter DNA-TBP and the promoter DNA-TBP-Bdp1SANT complexes. Data were accumulated from three independent measurements and data were fitted with a mono-exponential decay function to determine the complex lifetimes of 0.29 ± 0.01s for the DNA-TBP complex (light red) and 0.34 ±0.01 s for the DNA-TBP-Bdp1SANT sample (grey). (b) Representative dwell time histogram of the promoter DNA-TBP complex and (c) the promoter DNA-TBP-Bdp1SANT complex before normalization. (d) For the determination of the lifetime of the DNA-TBP-Brf2 and DNA-TBP-Brf2-Bdp1SANT samples, complexes were formed and freely diffusing single molecules were detected using confocal fluorescence microscopy. Complex dissociation was monitored via the relative amount of high FRET population after trapping the dissociated proteins with heparin. The resulting data were fitted with a mono-exponential decay function and lifetimes of 381 ±33 s for the DNA-TBP-Brf2 complex and an increased lifetime of 547 ± 87 s for the DNA-TBP-Brf2-Bdp1SANT complex were determined.

a

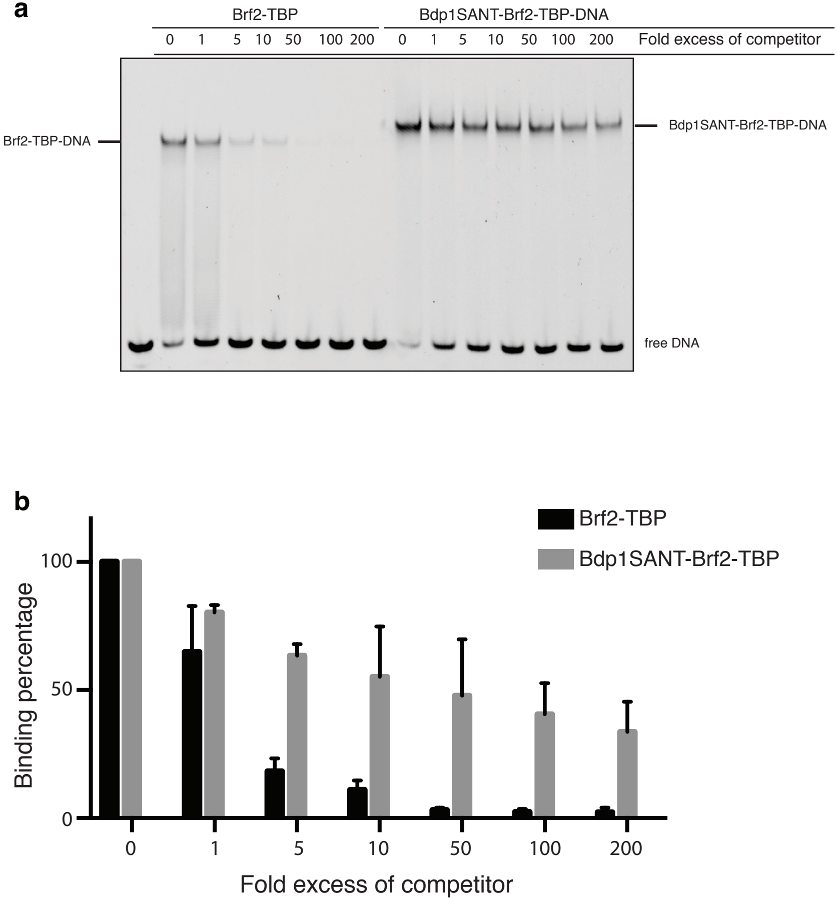

Supplementary Figure 7 EMSA competition assay of Brf2-TBP and Bdp1SANT-Brf2-TBP complexes. (**a**) A 0-200 molar excess of unlabelled DNA was incubated with pre-assembled complexes bound to labelled DNA. The molar excess of competitor is indicated on top of each lane. (**b**) Quantification of binding percentage is based on the intensity of the band corresponding to the bound complex, expressed as a fraction of the intensity of the band in absence of the unlabelled competitor (0 fold excess lanes). The error bar corresponds to the standard deviation.

Supplementary Table 1: FRET efficiencies [E] and percentage of dynamic molecules for the single-molecule FRET promoter bending essay control experiments. E is represented as the mean [x_c], standard deviation [σ] and standard error of a single or double Gaussian fit with the coefficient of determination [R^2]. Samples were first measured with the proteins in the flow chamber and again after washing away unbound proteins with buffer.

		protein in flow chamber	standard error	R^2	after washing	standard error	R^2
**Brf2**	**E_{low} [x_c ± σ]**	0.24 ± 0.06	0.001	0.9974			
	**E_{high} [x_c ± σ]**	-	0.003				
	**dyn. mol. [%]**	0					
**$Bdp1_{SANT}$**	**E_{low} [x_c ± σ]**	0.22 ± 0.05	0.002	0.9911			
	**E_{high} [x_c ± σ]**	-	-				
	**dyn. mol. [%]**	0					
**TBP $Bdp1_{SANT}$**	**E_{low} [x_c ± σ]**	0.24 ± 0.06	0.002	0.9870	0.23 ± 0.05	0.001	0.9951
	**E_{high} [x_c ± σ]**	0.54 ± 0.11	0.002		-	-	
	**dyn. mol. [%]**	36			2	-	
